# Editorial 2020

**DOI:** 10.4102/sajp.v76i1.1541

**Published:** 2020-12-17

**Authors:** Aimée V. Stewart

**Affiliations:** 1 Department of Physiotherapy, Faculty of Health Sciences, University of the Witwatersrand, Johannesburg, South Africa

As we were all celebrating the end of 2019 and the start of 2020 with all the promises of a new year and renewal, little did we know what was coming a few months later. Slowly but surely, the world became infected with the coronavirus disease 2019 (COVID-19), and countries in their efforts to control the virus, shut down their economies and people’s lives. The full impact of COVID-19 hit South Africans with the state of disaster, which started on 15th of March this year. Since then we have learned to live with the virus and everyone in all walks of life has learned to adapt. Learning went online, work went online, and we have learnt to live differently from the way we did before the pandemic.

As high-income countries like the United States of America and those in Europe battle a second wave of the virus, we hope that we will be spared a second wave with a high number of cases. Our current problems are to complete the academic year for those at schools, colleges and universities and to cope with a devastated economy. Our thoughts are continually with those who have lost their loved ones to the virus and those who have suffered so badly economically. We also keep all front-line healthcare workers and essential workers in our thoughts as they continue to keep the rest of us safe. I hope that all of us will continue with safe practices until such time as we come out of this pandemic.

The *South African Journal of Physiotherapy* (SAJP) has continued to thrive this year. For this, we owe a heartfelt thanks to our authors who have kept the submissions coming in; our reviewers who carefully consider the submissions and make appropriate recommendations and finally but not least our publishers who have often had to work remotely and who keep the wheels of the journal turning.

We are receiving more submissions from a greater variety of authors, not only from South Africa but slowly and surely from other countries as well. This is wonderful and we will continue to encourage this in all ways possible. The number of submissions has also increased and again we need to encourage this. The variety of topics published is increasing and there really should be something of interest for all our readers.

We have published two ‘state-of-the-art’ articles this year and are awaiting the promises of more that have been made by some of our senior researchers. We continually hope that we get bigger studies to publish and that researchers increasingly consider the SAJP as a journal of choice. To do this, we have to continue to improve the standard, and trust that by being on international indices our work is noticed by a larger audience.

This year we have also included additional checklists for authors, so that prior to submission they can check their articles to ensure they are at an acceptable level for scientific publication. The articles obviously still need to be reviewed, but we believe this additional checks will improve the standard of submissions in the long run. Now we also have more reviewers from Africa and are working continually to obtain even more.

So despite the hardships of working both remotely and in a difficult situation, the journal has thrived. We trust that the journal will continue to develop into the future as we constantly work on innovations to improve the quality of the SAJP.

Our best wishes for 2021 and stay safe and happy.

Prof. Aimée Stewart

Editor-in-Chief

*South African Journal of Physiotherapy*.

